# Treatment of pediatric alopecia areata: A systematic review

**DOI:** 10.1016/j.jaad.2021.04.077

**Published:** 2021-04-30

**Authors:** Virginia R. Barton, Atrin Toussi, Smita Awasthi, Maija Kiuru

**Affiliations:** a Department of Dermatology, University of California Davis, Sacramento; b Department of Pediatrics, University of California Davis, Sacramento; c Department of Pathology and Laboratory Medicine, University of California Davis, Sacramento.

**Keywords:** alopecia areata, contact immunotherapy, corticosteroids, JAK inhibitors, pediatric, quality of life

## Abstract

**Background:**

Alopecia areata (AA) is an autoimmune, nonscarring hair loss disorder with slightly greater prevalence in children than adults. Various treatment modalities exist; however, their evidence in pediatric AA patients is lacking.

**Objective:**

To evaluate the evidence of current treatment modalities for pediatric AA.

**Methods:**

We conducted a systematic review on the PubMed database in October 2019 for all published articles involving patients <18 years old. Articles discussing AA treatment in pediatric patients were included, as were articles discussing both pediatric and adult patients, if data on individual pediatric patients were available.

**Results:**

Inclusion criteria were met by 122 total reports discussing 1032 patients. Reports consisted of 2 randomized controlled trials, 4 prospective comparative cohorts, 83 case series, 2 case-control studies, and 31 case reports. Included articles assessed the use of aloe, apremilast, anthralin, anti-interferon gamma antibodies, botulinum toxin, corticosteroids, contact immunotherapies, cryotherapy, hydroxychloroquine, hypnotherapy, imiquimod, Janus kinase inhibitors, laser and light therapy, methotrexate, minoxidil, phototherapy, psychotherapy, prostaglandin analogs, sulfasalazine, topical calcineurin inhibitors, topical nitrogen mustard, and ustekinumab.

**Limitations:**

English-only articles with full texts were used. Manuscripts with adult and pediatric data were only incorporated if individual-level data for pediatric patients were provided. No meta-analysis was performed.

**Conclusion:**

Topical corticosteroids are the preferred first-line treatment for pediatric AA, as they hold the highest level of evidence, followed by contact immunotherapy. More clinical trials and comparative studies are needed to further guide management of pediatric AA and to promote the potential use of pre-existing, low-cost, and novel therapies, including Janus kinase inhibitors.

Alopecia areata (AA) is a nonscarring hair loss disorder that affects up to 2% of the global population.^1^ It is estimated that nearly 80% of patients with limited, patchy AA spontaneously recover.^2^ AA is characterized by relapsing and remitting patches of hair loss that may progress to severe subtypes, such as alopecia totalis (AT), alopecia universalis (AU), or alopecia ophiasis (AO), often resulting in significant psychological detriment. The pediatric population is particularly susceptible to the psychosocial consequences of AA, thus, adequate treatment is critical to prevent further morbidity associated with this disease.^3^ Although there are currently no treatments for AA approved by the Food and Drug Administration, there are numerous off-label treatment options for adults with AA. Therapeutic options for children and adolescents are limited. This systematic review sought to evaluate available off-label therapies for the treatment of AA in patients younger than 18 years of age.

## METHODS

A systematic review was conducted according to the Preferred Reporting Items for Systematic Review and Meta-Analyses (PRISMA) guidelines (Supplemental Table I; available via Mendeley at https://doi.org/10.17632/s9rx4myvnn.1). Using the PubMed database, a search for all published peer-reviewed articles was performed using the following search terms: ‘‘alopecia’’ and ‘‘areata’’ or ‘‘totalis’’ or ‘‘universalis’’ or ‘‘ophiasis’’ and ‘‘treatment’’ or ‘‘therapy’’ or ‘‘medication’’ or ‘‘drug.’’

These records were screened using defined criteria for eligibility, which consisted of English articles discussing the direct study or report of treatment modalities for AA in humans younger than 18 years of age. References of included reports were examined and additional sources not identified initially were incorporated. Review articles, animal studies, articles evaluating treatments that are no longer manufactured worldwide, including alefacept, and articles with unavailable full text were excluded. Articles that reported on results for both pediatric and adult patients were only included if individual-level data for the pediatric patients were provided.

The results were then further classified by the Oxford Centre for Evidence-Based Medicine 2011 Levels of Evidence (LoE): level 1 (systematic review of randomized controlled trials [RCTs] or high-quality randomized controlled trial), level 2 (lesser quality RCTor prospective cohort study), level 3 (case-control study, non-randomized controlled cohort or follow-up study), level 4 (case series), or level 5 (expert opinion, mechanism-based reasoning).

## RESULTS

**Fig 1.**
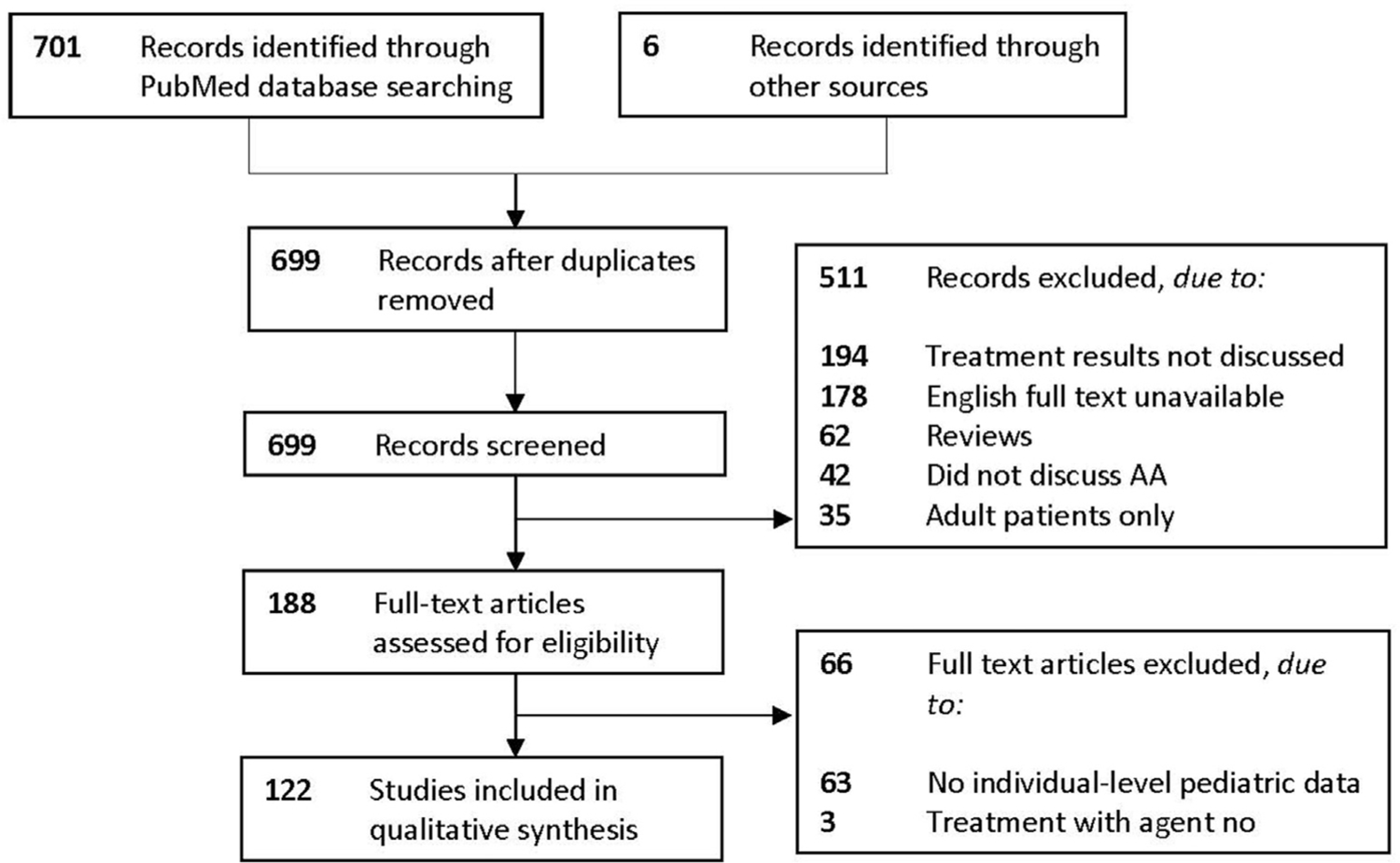
PRISMA 2009 flow diagram illustrating a total of 707 publications retrieved, of which 122 reports were included. AA, Alopecia areata; *PRISMA*, Preferred Reporting Items for Systematic Review and Meta-Analyses.

A total of 707 publications were retrieved, of which 122 reports were included (Fig 1). These reports consisted of 2 RCTs, 4 prospective comparative cohorts, 83 case series, 2 case-control studies, and 31 case reports. Included articles and results are summarized in Tables I to III.^4–18^

### Topical therapies

#### Anthralin.

Useof the irritant anthralin to treat AA in pediatric patients was demonstrated in 4 case series or reports, including 69 patients (strongest LoE 4; Table I).^19–22^ Complete response rates ranged between 32% and 33.3% with relapse rates of 9.5% to 64%. One case reported complete regrowth when combined with leflunomide.^19^ The mean time to maximal response was approximately 9 to 15 months.^19–22^ Anthralin caused staining of the skin and regional lymphadenopathy (LAD), which resolved after cessation of treatment. Other side effects were itching, burning, oozing, and bullous eruptions, but systemic side effects were rare.^118^

#### Contact immunotherapy.

##### Diphenylcyclo propenone.

Treatment of the affected areas with diphenylcyclopropenone (DPCP) includes sensitization prior to initial treatment and escalating dose concentrations. The essentially painless application method makes DPCP an ideal and frequently utilized treatment option for the pediatric population. Eight articles reported DPCP treatment in 200 children with AA (strongest LoE 3).^23–30^ Complete response rates ranged from 0% to 33.3%, similar to the results of a meta-analysis (30.7%).^119^ Relapses were common, with relapse rates ranging from 12.5% to 58.3%.^28,29,30^ One case-control study noted the potential of imiquimod to improve DPCP efficacy.^23^ Side effects included eczematous reactions of the scalp, pruritus, regional LAD, vesiculation, or, rarely, a secondary infection.^29^ No systemic side effects except headache were reported.

##### Squaric acid dibutyl ester.

The efficacy of squaric acid dibutyl ester (SADBE) was studied in 78 pediatric patients (strongest LoE 4). Complete response rates ranged from 0% to 33.3%.^33–35^ A meta-analysis including adult and pediatric patients demonstrated slightly better complete response rates with SADBE (38.4%) than with DPCP (30.7%).^119^ Relapse rates ranged between 62.5% and 100%. Side effects included irritation, itching, LAD, and contact dermatitis.^31^ There was 1 case of epidermolysis bullosa aquisita that arose during treatment of AA with SADBE and regressed upon discontinuation.^32^ There was no evidence of systemic absorption through topical application.^120^

#### Cryotherapy.

One case series documented the use of cryotherapy in 24 patients <10 years of age and 40 patients between the ages of 10 and 20 (strongest LoE 4). Complete response was seen in 20.8% of patients <10 years of age. Side effects were localized, but included pain, pruritus, inflammation, and swelling.^36,121^

#### Minoxidil.

Minoxidil’s efficacy is equivocal for adult AA^122^ and only case reports exist evaluating its use in 9 children (strongest LoE 4). Minoxidil is mostly used as an adjunctive therapy.^41,83^ Side effects of minoxidil included extensive hypertrichosis.^37–40,42^ Although excessive topical administration may lead to systemic absorption (manifesting as palpitations, hypotension, etc.), the typical twice daily dose is generally safe.^123^

#### Topical calcineurin inhibitors.

The consensus of 4 studies that included 7 pediatric AA patients is that topical calcineurin inhibitors, tacrolimus and pimecrolimus, are not effective for the treatment of AA (strongest LoE 2). Approximately 29% showed only a minimal response,^44^ while the remaining 71% showed no response and often experienced disease progression.^45–47,124^

#### Topical and intralesional corticosteroids.

The use of topical corticosteroids, particularly high-potency topical corticosteroids, is supported by the literature (strongest LoE 1) and is considered a safe and effective first-line treatment option in children with patchy AA. High-potency topical corticosteroids showed a higher efficacy than low-potency topical corticosteroids in a RCT that included 41 pediatric patients.^50^ They were also superior to topical tacrolimus^44^ and anthralin^22^ and were often used as adjunctive therapies.^49,51,63,83^ High-potency topical corticosteroids were generally well tolerated in children. Side effects included skin atrophy, telangiectasias, and folliculitis. Although intralesional corticosteroid (triamcinolone) therapy is effective, these studies are rare in children due to the pain associated with the injections.^48^ Based on data on adult patients, the most common side effects are pain, skin atrophy, and dyspigmentation. Other adverse effects are rare, although anaphylaxis and cataracts and increased intraocular pressure, if used close to the eyes, have been reported.^125^

#### Prostaglandins.

Topical prostaglandins, including bimatoprost and latanoprost, may improve the regrowth of scalp and eyelash hair (strongest LoE 1–2) in AA,^52–56^ although statistically significant differences between bimatoprost and vehicle were not found in a RCT examining eyelash hair growth in pediatric AA patients.^52^ While prostaglandins, specifically latanoprost, can cause irreversible iris and eyelid hyperpigmentation, uveitis, eyelash curling, and conjunctival hyperemia, these side effects were not reported in patients with AA.^52–56,126^

### Systemic therapies

#### Corticosteroids.

Systemic corticosteroid therapy was the most studied treatment modality for AA in both children and adults, comprising 27 studies, mostly case series, that included 272 pediatric patients (strongest LoE 2; Table II). The studies included combination therapy with an adjunctive systemic drug including methotrexate or cyclosporine,^60–62,68,72^ intravenous pulse-dosed corticosteroids,^68,70–74,77,79,81,82^ oral pulse-dosed corticosteroids,^49,60,69,71,75,76,78,80^ oral corticosteroid maintenance or tapered therapy,^61,62,64–67^ and intramuscular corticosteroids.^57–59^

Although doses and frequencies varied among each route of administration, approximately 45% (range 0% to 100%) of patients receiving intravenous or oral pulse-dosed corticosteroids demonstrated a complete response and 34% (range 0% to 55.5%) of patients receiving traditional oral corticosteroid regimens demonstrated a complete response. For pulse-dosed therapy, shorter disease duration, younger age at disease onset, and multifocal disease (as opposed to AT and AU) were found to be associated with a better response.^71^ Relapse rates ranged between 16.7 and100% for pulse-dosed and 50% and 100% for non-pulse-dosed corticosteroids.^59,64^ Significant side effects were reported, including weight gain, cataracts, infections, hypertension, Cushingoid features, psychiatric disturbances, striae, and acne. Side effects were greater and more frequent for non-pulse-dosed regimens (Table II).^127,128^

#### Hydroxychloroquine.

A single case series of 9 pediatric patients examined the use of hydroxychloroquine (strongest LoE 4). When used in conjunction with topical corticosteroids and/or minoxidil, complete response was seen in 11% and partial response in 55% of patients.^83^ Reported side effects included abdominal pain and headache.^83^

#### Methotrexate.

Eight articles reported studies of methotrexate, either as a solitary agent or in conjunction with oral or intravenous corticosteroids or azathioprine, for the treatment of AA in 42 pediatric patients (strongest LoE 4).^60,68,72,84–88^ Complete response was seen on average in 17.9% (range 0% to 50%; Table II) and partial response in 47.9% (range 0% to 100%) with doses ranging from 2.5 mg to 25 mg per week.^60,72,85–88^ A meta-analysis revealed a higher complete response in adult versus pediatric AA patients (44.7% vs 11.6%), although the relapse rate in children was significantly lower than that in adults (31.7% vs 52%).^129^ Reported side effects included nausea, elevations in hepatic transaminases, and hematologic changes (Table II).

#### Sulfasalazine and mesalazine.

Limited data exist for the use of sulfasalazine and mesalazine for pediatric AA (strongest LoE 4). Complete response to mesalazine, with or without concurrent oral or topical corticosteroids and minoxidil, was reported in 1 case series of 5 pediatric patients.^89^ Ten adolescent AA patients treated with oral sulfasalazine in 2 studies all demonstrated partial response with a starting dose of 1 g/week, which was escalated to a final dose of 3 g/week.^90,91^ Side effects for sulfasalazine included dizziness, headache, and dyspepsia (Table II). This was similar to the side-effect profile in adults, which included gastrointestinal distress, rash, headache, and lab abnormalities.^130^

#### Ustekinumab.

A report of 3 adults whose AA responded to ustekinumab, a monoclonal antibody used for psoriasis that blocks interleukins 12 and 23,^131^ prompted the treatment in pediatric AA and AT patients with variable results (strongest LoE was 4). One case series showed a complete or partial response in all 3 patients, while the other study reported no response.^92,93^ Although injection-site reactions, infections, nausea, and vomiting are known side effects of ustekinumab, none were reported in these 2 studies.

#### Janus kinase inhibitors.

Increasing evidence suggests that JAK inhibitors may be effective in the treatment of AA, but data in children are limited (strongest LoE 4). Side effects included infections, diarrhea, hypertension, thrombosis, gastrointestinal perforation, laboratory abnormalities, and hematologic malignancies.^132^

##### Baricitinib.

Clinical trials have been initiated to evaluate the safety and efficacy of baricitinib for the treatment of AA in adults but not yet in children.^133,134^ Only 1 pediatric case has been reported (strongest LoE 5). A 17-year-old male with a longstanding history of recalcitrant AA initially showed a partial response with baricitinib 7 mg once daily, followed by a complete response when the dose was increased to 11 mg once daily.^94^ No relapse was reported.

##### Ruxolitinib.

A case series of 8 AA patients treated with ruxolitinib included only 1 pediatric patient, who was treated with ruxolitinib 10 mg twice daily for 10 months and experienced a 91% improvement in the Severity of Alopecia Tool score with no adverse events.^101^

##### Tofacitinib.

Clinical trials are currently evaluating the efficacy of tofacitinib to treat AA in adults.^99^ Six case series and reports evaluated systemic tofacitinib for the treatment of AA in 28 pediatric patients.^95–100^ Of these patients, 82% showed complete or partial response and all nonresponders were patients with AU. Similarly, adults with severe AT or AU present for >10 years were less likely to respond to tofacitinib.^100^ Side effects included diarrhea, headaches, upper respiratory infection, increased appetite, weight gain, fatigue, and transient elevation in transaminases.

##### Topical tofacitinib and ruxolitinib.

In 3 reports documenting a total of 18 pediatric patients, 13 responded to topical therapy.^102–104^ Side effects included application site irritation^102^ and 1 case of borderline leukopenia in a patient with baseline low white blood cell count.^104^

### Laser and phototherapy

#### Laser therapy.

Seventeen patients received treatment with a 308 nm excimer laser twice weekly with 58.8% response rate (strongest LoE 4).^105–108^ Side effects included mild scalp erythema and desquamation.

#### Phototherapy.

There were 6 reports involving 26 pediatric AA patients treated with psoralen and ultraviolet A therapy (strongest LoE 4).^110–115,117^ All 5 adolescents treated with a psoralen-soaked towel followed by sun exposure demonstrated partial response.^116^ Narrow-band ultraviolet B therapy was largely ineffective in pediatric patients,^109^ similar to the results in adults.^135^ Mild irritation, erythema, pruritus, and scaling were noted as side effects of phototherapy, similar to adult patients with AA.^116^

## DISCUSSION

AA is an immune-mediated disease causing non-scarring hair loss with significant psychosocial impact.^1^ While a majority of children with limited AA spontaneously recover, the variability of the disease course and unpredictable response to therapy make AA challenging to treat. Although numerous therapies have been reported, the evidence is mostly weak. As a general guideline, low-risk topical therapies are a reasonable option for limited AA, while higher-risk systemic therapies may be warranted for patients who have extensive AA refractory to other therapies and who experience a significant psychosocial impact.

A limited number of trials have been conducted in pediatric AA patients, mostly involving topical corticosteroids.^44,50^ These studies provide the highest LoE for treatment with high-potency topical corticosteroids and have led to their preference as first-line therapy for pediatric AA. While intralesional corticosteroids are recommended as first-line treatment for patchy AA in adults,^136^ their use in children is limited by pain.^137^ Systemic steroids also can be efficacious, particularly in patients with a shorter disease duration, those who are at a younger age at disease onset, and those with multifocal disease^71^; however, their use is limited by significant side effects.^127,128^

Other treatment options include contact immunotherapy with DPCP or SADBE, although evidence in children is limited to case series^24–30,33–35^ (Table I). Protocols for the application of SADBE at home have been utilized more recently, increasing its convenience but increasing out-of-pocket cost when purchasing SADBE from compounding pharmacies. With respect to topical adjuvant therapy, minoxidil is commonly used as the ‘‘go-to’’ secondary agent in clinical practice, but our evidence does not support its use as a first-line agent^122^ (Table I). Topical calcineurin inhibitors are ineffective.^45–47,124^

A better understanding of the molecular pathogenesis of AA has resulted in the development of targeted therapies, including JAK inhibitors. Current clinical trials for adults with AA include treatment with tofacitinib, ruxolitinib, and baricitinib.^133^ Furthermore, clinical trials have been initiated recently to evaluate a JAK inhibitor, PF-06651600, for AA treatment in adults and adolescents older than 12 years of age.^133^ If pediatric data are able to reflect preliminary adult responses to systemic JAK inhibitors, these currently show promise as potential future therapies, but more trials, including trials with pediatric patients, are needed. While systemic JAK inhibitors may be an effective new therapy, their safety profile as well as cost may significantly limit their use to severe, treatment-refractory cases.^99,132^

It is also important to counsel patients and families regarding the chronicity of AA and the relapsing and remitting nature of the disease. Because of the lack of an evidence-based treatment algorithm, we recommend counseling patients and their families on the wide range of severity and varied responses to treatment among the different AA subtypes. Specifically, most data on AA are generalized from heterogenous groups of individuals, including patients with AT and AU. Subtype-specific response to treatment is not well-documented; however, it is known that the AT and AU subtypes generally bode more recalcitrant disease and worse outcomes. Clinicians should also highlight the existence and impact of AA comorbidities, particularly co-occurring autoimmune conditions, such as vitiligo, which add to the psychosocial impact of an AA diagnosis and can have long-lasting effects on self-esteem during childhood.^138^

## CONCLUSIONS

Pediatric AA has a variable disease course with significant psychosocial impact. Although topical corticosteroids remain the preferred first-line treatment for pediatric AA, RCTs, and prospective comparative studies are needed to help define treatment guidelines. Additionally, a better understanding of prognostic markers in AA would be valuable.

## Supplementary Material

Supplemental_Table_1

## Figures and Tables

**Table I.** Included studies evaluating topical and miscellaneous treatment of alopecia areata in pediatric patients

First author	Year	Treatment	LoE	Study type	N	AA	AT	AU	AO	CR*	PR^†^	NR^‡^	RR^§^	SE
Anthralin														
Sardana^19^	2018	Anthralin + leflunomide	5	Case report	1	-	-	-	1	1 (100%)	-	-	NA	Itching, burning
Wu^20^	2018	Anthralin	4	Case series	37	24	8	3	2	12 (32%)	15 (40%)	5 (14%)	16 (64%)	Irritation, LAD
Ozdemir^21^	2017	Anthralin	4	Case series	30	27	1	2	-	10 (33.3%)	11 (36.7%)	9 (30%)	2 (9.5%)	Irritation, itching, LAD, hyperpigmentation, crusting, oozing, bullous eruption
Torchia^22^	2015	Anthralin + TC	5	Case report	1	1	-	-	-	-	-	1 (100%)	NA	LAD
Contact														
Immunotherapy														
Wasylyszyn^23^	2016	DPCP + imiquimod vs DPCP	3	Case-control	9	1	3	5	-	Both-2/3 (66.7%) DPCP only-0/6 (0%)	Both-1/3 (33.3%) DPCP only-2/6 (33.3%)	Both-0/3 (0%) DPCP only-4/6 (66.7%)	NA	Scalp eczema, discomfort, LAD
Luk^24^	2012	DPCP	4	Case series	3	-	2	1	-	-	-	3 (100%)	NA	Itching, erythema, bulla, scaling, LAD, hyperpigmentation, urticarial reactions
Salsberg^25^	2012	DPCP	4	Case series	108	82	-	-	26	12 (11%)	23 (21%)	27 (25%)	NA	Edema, dermatitis, vesicles, desquamation, urticaria, erosions, LAD
Singh^26^	2007	DPCP	4	Case series	3	-	-	-	3	1 (33.3%)	2 (66.7%)	-	NA	None
Sotiriadis^27^	2006	DPCP	4	Case series	14	7	3	4	-	2 (14.3%)	8 (57.1%)	4 (28.6%)	NA	Eczema, headache, itching, hyperpigmentation
Schuttelaar^28^	1996	DPCP	4	Case series	25	10	15	-	-	8 (32%)	4 (16%)	13 (52%)	7 (58.3%)	Eczema, itching, vesicles, headache, LAD
Hull^29^	1991	DPCP	4	Case series	12	4	8	-	-	4 (33.3%)	4 (33.3%)	4 (33.3%)	4 (50%)	Eczema with superimposed infection, blistering
Orecchia^30^	1985	DPCP	4	Case series	26	9	7	10	-	1 (3.8%)	13 (50%)	12 (46.1%)	4 (28.6%)	LAD, itching, eczema
Chen^31^	2017	SADBE	5	Case report	1	1	-	-	-	-	1 (100%)	-	NA	Angioedema
Guerra^32^	2017	SADBE	5	Case report	1	1	-	-	-	-	1 (100%)	-	1 (100%)	Epidermolysis bullosa acquisita
Tosti^33^	1996	SADBE	4	Case series	33	-	10	23	-	10 (30.3%)	6 (18.2%)	17 (51.5%)	10 (62.5%)	Contact dermatitis, LAD
Orecchia^34^	1995	SADBE	4	Case series	28	NA	NA	NA	NA	9 (32.1%)	6 (21.4%)	13 (46.4%)	NA	None
Giannetti^35^	1983	SADBE	4	Case series	15	NA	NA	NA	NA	1 (6.6%)	6 (40%)	8 (53.3%)	NA	Eczema, LAD, itching
Cryotherapy														
Jun^36^	2017	Cryotherapy	4	Case series	24	NA	NA	NA	NA	5 (20.8%)	15 (62.5%)	4 (16.7%)	NA	Pain, pruritus, inflammation, swelling
Minoxidil														
Rai^37^	2017	Minoxidil	5	Case report	1	1	-	-	-	-	-	1 (100%)^ǁ^	NA	Hypertrichosis
Guerouaz^38^	2014	Minoxidil	5	Case report	1	1	-	-	-	-	1 (100%)^ǁ^	-	NA	Hypertrichosis
Herskovitz^39^	2013	Minoxidil	5	Case report	1	1	-	-	-	-	1 (100%)^ǁ^	-	NA	Hypertrichosis
Georgala^40^	2007	Minoxidil	4	Case series	3	2	1	-	-	-	-	3 (100%)^ǁ^	NA	Palpitations, dizziness, sinus tachycardia
Lenane^41¶^	2005	Minoxidil	4	Case series	1	-	1	-	-	-	-	1 (100%)^ǁ^	NA	None
Baral^42#^	1989	Minoxidil + TC + ILC	5	Case report	1	1	-	-	-	-	1 (100%)^ǁ^	-	NA	Hypertrichosis
Weiss^43^	1981	Minoxidil	4	Case series	1	-	-	1	-	-	1 (100%)	-	NA	None
Topical Calcineurin Inhibitors														
Jung^44^	2017	Topical tacrolimus vs clobetasol, split-scalp	2	Prospective comparative cohort	3	3	-	-	-	TC-2/3 (66.7%) TT-0/3 (0%)	TC-1/3 (33.3%) TT-2/3 (66.7%)	TC-0/3 (0%) TT-1/3 (33.3%)	NA	None
Rigopoulos^45^	2007	Topical pimecrolimus vs placebo, split-scalp	2	Prospective comparative cohort	1	1	-	-	-	-	-	1 (100%)	NA	Burning
Price^46^	2005	Topical tacrolimus	4	Case series	2	2	-	-	-	-	-	2 (100%)	NA	None
Thiers^47^	2000	Topical tacrolimus	5	Case report	1	1	-	-	-	-	-	1 (100%)	NA	NA
Topical and Intralesional Corticosteroids														
Sankararaman^48^	2017	ILC	5	Case report	1	-	-	-	1	-	1 (100%)	-	1 (100%)	None
Jung^44^	2017	Clobetasol vs topical tacrolimus, split-scalp	2	Prospective comparative cohort	3	3	-	-	-	TC-2/3 (66.7%) TT-0 (0%)	TC-1/3 (33.3%) TT-2/3 (66.7%)	TC-0/3 (0%) TT-1/3 (33.3%)	NA	None
Lalosevic^49#^	2015	Oral PDC + clobetasol	4	Case series	65	35	15	15	26 (40%)	17 (26.2%)	22 (33.8%)	11 (25.6%)	Headache (after oral PDC), skin atrophy	
Torchia^22^	2015	Triamcinolone + clobetasol vs anthralin, split-scalp	5	Case report	1	1	-	-	-	TC side	-	Anthralin side	NA	None
Lenane^50^	2014	Clobetasol vs hydrocortisone	1	Randomized controlled trial	41	41	-	-	-	>50% regrowth Clobetasol-17/20 (85%) Hydrocortisone-7/21 (33.3%)	<50% regrowth Clobetasol-3/20 Hydrocortisone-14/21 (66.7%)		NA	Skin atrophy
Lenane^41¶^	2005	TC	4	Case series	4	2	2	-	-	2 (50%)	1 (25%)	1 (25%)	1 (50%)	Skin atrophy
Baral^42#^	1989	Minoxidil + TC + ILC	5	Case report	1	1	-	-	-	-	-	1 (100%)^ǁ^	NA	Hypertrichosis
Montes^51^	1977	Halcinonide	4	Case series	2	1	1	-	-	2 (100%)	-	-	NA	Folliculitis
Prostaglandins														
Borchert^52^	2016	Bimatoprost	1/2	Randomized controlled trial	15	NA	NA	NA	NA	-	Bimatoprost-5/9 (55.6%); Vehicle-1/6 (16.7%)	Bimatoprost-4/9 (44.4%); Vehicle-5/6 (83.3%)	NA	Conjunctival hyperemia, conjunctivitis, eczema, eyelid erythema
Li^53^	2016	Bimatoprost (scalp)	5	Case report	1	1	-	-	-	1 (100%)	-	-	NA	None
Zaheri^54^	2010	Bimatoprost	5	Case report	1	1	-	-	-	1 (100%)	-	-	NA	None
Yadav^55^	2009	Latanoprost	5	Case report	1	1	-	-	-	1 (100%)	-	-	NA	None
Mehta^56^	2003	Latanoprost	5	Case report	1	1	-	-	-	-	1 (100%)	-	NA	None

*AA*, Alopecia areata; *AO*, alopecia ophiasis; *AT*, alopecia totalis; *AU*, alopecia universalis; *CR*, complete response; *DPCP*, diphenylcyclopropenone; *ILC*, intralesional corticosteroids; *LAD*, lymphadenopathy; *LoE*, level of evidence; *N*, number of pediatric patients; *NA*, not available; *NR*, no response; *OC*, oral corticosteroids; *PDC*, pulse dose corticosteroids; *PR*, partial response; *PT*, psychotherapy; *RR*, relapse rate; *SADBE*, squaric acid dibutylester; *SE*, side effects; *TC*, topical corticosteroids; *TT*, topical tacrolimus.

* Complete response defined as $95% hair regrowth, (n %) = percent of total number of patients.

† Partial response defined as\95% and[0% hair regrowth, (n %) = percent of total number of patients.

‡ No response defined as 0% hair regrowth, (n %) = percent of total number of patients.

§ Relapse rate defined as number of patients who responded to treatment and experienced recurrence of hair loss, (n %) = percent of responsive patients.

|| Patient(s) discontinued study due to adverse events.

¶ Study listed under both Minoxidil and TC sections as it provides data for both treatments in separate patients.

# Study listed under multiple sections due to inclusion of multiple treatments.

**Table II.** Included studies evaluating systemic treatment of alopecia areata in pediatric patients

First author	Year	Treatment	LoE	Study type	N	AA	AT	AU	AO	CR*	PR^†^	NR^‡^	RR^§^	SE
Intramuscular Corticosteroids														
Seo^57^	2017	IMC	4	Case series	2	-	2	-	-	1 (50%)	1 (50%)	-	NA	None
Sato-Kawamura^58^	2002	IMC	4	Case series	1	-	1	-	-	1 (100%)	-	-	NA	None
Michalowski^59^	1978	IMC	4	Case series	6	-	5	1	-	2 (33.3%)	2 (33.3%)	2 (33.3%)	4 (100%)	Hypertrichosis, diabetes, moon facies, striae, dysmenorrhea, pseudoacanthosis nigricans^ǁ^
Oral Corticosteroids														
Anuset^60#^	2016	OC + MTX	4	Case series	4	1	1	2	-	2 (50%)	-	2 (50%) (1 on MTX only)	2 (100%)	Transient elevation of transaminases, weight gain, cataracts, pneumocystis pneumonia^ǁ^
Gensure^61^	2013	OC + cyclosporine	5	Case report	1	-	1	-	-	1 (100%)	-	-	NA	Confluent and reticulated papillomatosis
Kim^62^	2008	OC + cyclosporine	4	Case series	9	5	4	-	-	5 (55.5%)	3 (33.3%)	1 (11.1%)	NA	Edema, acne, weight gain, hypertrichosis, GI disturbance, menstrual abnormality
Camacho^63^	1999	OC vs ZBC	2	Prospective comparative cohort	18	6	12	3	-	OC-0/9 (0%) ZBC-3/9 (33.3%)	OC-4/9 (44.4%) ZBC-5/9 (55.5%)	OC-5/9 (55.5%) ZBC-1/9 (11.1%)	NA	Cushingoid features, delayed physical development
Alabdulkareem^64^	1998	OC	4	Case series	11	-	8	1	-	1 (9%)	5 (45.4%)	5 (45.4%)	5 (83.3%)	Acne, striae, moon facies
Schindler^65^	1987	OC	5	Case report	1	-	-	1	-	1 (100%)	-	-	0 (0%)	Weight gain, Cushingoid features
Unger^66^	1978	OC	4	Case series	6	1	4	1	-	3 (50%)	3 (50%)	-	3 (50%)	Weight gain
Winter^67^	1976	OC	4	Case series	12	3	4	5	-	5 (41.7%)	-	7 (58.3%)	NA	Weight gain, abdominal pain, cataracts, acne, hypertension, seizure, psychological problems, obesity
Pulse Dose Corticosteroids														
Chong^68#^	2017	IV PDC + MTX	4	Case series	14	-	14		-	1 (7.1%)	5 (35.7%)	8 (57.1%)	NA	Abdominal discomfort
John-Bassler^69^	2017	IV PDC	4	Case series	13	6	5	2	-	8 (61.5%)	-	5 (38.5%)	3 (37.5%)	Weight gain, acne
Lalosevic^49#^	2015	Oral PDC + TC	4	Case series	65	35	15		15	26 (40%)	17 (26.2%)	22 (33.8%)	11 (25%)	Headache, skin atrophy
Smith^70^	2015	IV PDC	4	Case series	18	2	2	3	11	2 (11.1%)	9 (50%)	7 (38.9%)	7 (63.6%)	Mood changes, metallic taste, acne, allergic reaction
Friedland^71^	2013	IV PDC	4	Case series	24	8	4	1	10	9 (37.5%); 5/8 AA, 1/4 AT, 0/2, AU, 3/10 AO	7 (29.2%); 1/8 AA, 1/4 AT, 1/2, AU, 4/10 AO	8 (33.3%); 2/8 AA, 2/4 AT, 1/2, AU, 3/10 AO	13 (81.2%); 5/6 AA, 1/2 AT, 1/1, AU, 6/7 AO	Verrucae, gastritis, abdominal pain
Droitcourt^72#^	2012	IV PDC + MTX	4	Case series	2	2	-	-	-	1 (50%)	1 (50%)	-	2 (100%)	Nausea, neutropenia
Sauerbrey^73^	2011	IV PDC + TT	4	Case series	2	-	1	-	-	2 (100%)	-	-	1 (50%)	None
Hubiche^74^	2008	IV PDC	4	Case series	12	-	4	1	7	-	10 (83.3%)	2 (16.7%)	6 (60%)	None
Sethuraman^75^	2006	Oral PDC + minoxidil	5	Case report	1	-	-	1		-	1 (100%)	-	NA	None
Bin Saif^76^	2006	Oral PDC	5	Case report	1	-	-	1	-	1 (100%)	-	-	1 (100%)	Nocturnal enuresis
Seiter^77^	2001	IV PDC	4	Case series	4	2	1	1	-	2 (50%); 2/2 AA, 0/1 AT, 0/1 AU	-	2 (50%)	NA	Headache, fatigue, nausea, palpitations
Sharma^78^	1999	Oral PDC	4	Case series	4	NA	NA	NA	NA	4 (100%)	-	-	NA	NA
Friedli^79^	1998	IV PDC	4	Case series	7	1	4	1	1	1 (14.3%); 1/1 AA, 0/4 AT, 0/1 AU, 0/1 AO	2 (28.6%); AA 0/1, AT 1/4, AU 0/1, AO 1/1	4 (57.1%); AA 1/1, 3/4 AT, AU 1/1, AO 0/1	2 (66.7%); AA 0/1, AT 1/1, 1/1 AO	Fatigue, headache, palpitations, dyspnea, nausea
Sharma^80^	1998	Oral PDC	4	Case series	16	13	3	-	1	6 (37.5%)	6 (37.5%)	3 (18.7%)	4 (33.3%)	Epigastric burning, headache
Kiesch^81^	1997	IV PDC	4	Case series	7	3	1	-	3	5 (71.4%); AA 3/3, AO 2/3	-	2 (28.6%); AT 1/1, AO 1/3	1 (20%)	Abdominal pain
Perriard-Wolfensberger^82^	1993	IV PDC	4	Case series	1	1	-	-	-	-	1 (100%)	-	NA	Flushing
Hydroxychloroquine														
Yun^83^	2018	HCQ +/− TC and/or minoxidil	4	Case series	9	6	1	2	-	1 (11.1%)	5 (55.5%)	3 (33.3%)	NA	Headache, abdominal pain, viral gastroenteritis
Methotrexate														
Mascia^84^	2019	MTX + azathioprine	4	Case series	3	2	1	-	-	-	3 (100%)	-	NA	GI distress, lymphopenia^ǁ^
Chong^68#^	2017	MTX + IV PDC	4	Case series	14	-	14		-	1 (7.1%)	5 (35.7%)	8 (57.1%)	NA	Abdominal discomfort
Landis^85^	2018	MTX	4	Case series	11	NA	NA	NA	NA	4 (36.4%)	7 (63.6%)	-	2 (18.1%)	Leg weakness, tooth sensitivity
Anuset^68¶^	2016	MTX + OC	4	Case series	4	1	1	2	-	2 (50%)	-	2 (50%) (1 on MTX only)	2 (100%)	Transient elevation of transaminases, weight gain, cataracts, pneumocystis pneumonia^ǁ^
Batalla^86^	2016	MTX	4	Case series	3	1	1	-	1		2 (66.7%)	1 (33.3%)	1 (50%)	Elevated hepatic transaminases
Lucas^87^	2016	MTX	4	Case series	13	NA	NA	NA	NA	-	5 (38.5%)	8 (61.5%)	2 (40%)	Recurrent nausea
Droitcourt^72#^	2012	MTX + IV PDC	4	Case series	2	2	-	-	-	1 (50%)	-	-	2 (100%)	Nausea, neutropenia
Royer^88^	2011	MTX +/− OC	4	Case series	14	7	7		-	-	11 (78.6%)	3 (21.4%)	3 (27.3%)	Nausea, herpes zoster
Sulfasalazine and Mesalazine														
Kiszewski^89^	2018	Mesalazine +/− TC, OC, minoxidil	4	Case series	5	3	-	1	1	5 (100%)	-	-	NA	None
Rashidi	2008	Sulfasalazine	4	Case series	7	4	3	-	-	-	7 (100%)	-	NA	Dizziness, headache, dyspepsia
Bakar^91^	2007	Sulfasalazine+ OC	4	Case series	3	3	-	-	-	-	3 (100%)	-	NA	None
Ustekinumab														
Aleisa^92^	2019	Ustekinumab	4	Case series	3	2	1	-	-	1 (33.3%)	2 (66.7%)	-	NA	NA
Ortolan^93^	2019	Ustekinumab	4	Case series	3	-	3	-	-	-	-	3 (100%)	NA	NA
JAK Inhibitors														
Jabbari^94^	2015	Baricitinib	5	Case report	1	1	-	-	-	1 (100%)	-	-	NA	None
Craiglow^95^	2019	Tofacitinib	4	Case series	4	-	1	3	-	2 (50%)	1 (25%)	1 (25%)	NA	None
Dai^96^	2019	Tofacitinib	4	Case series	3	-	2	1	-	1 (33.3%)	2 (66.7%)	-	NA	Diarrhea, URI
Brown^97^	2018	Tofacitinib	5	Case report	1	-	-	1	-	1 (100%)	-	-	NA	Headache
Patel^98^	2018	Tofacitinib	4	Case series	1	-	-	1	-	-	1 (100%)	-	NA	Increased appetite, weight gain
Castelo-Soccio^99^	2017	Tofacitinib	4	Case series	6	-	-	6	-	-	6 (100%)	-	NA	None
Craiglow^100^	2017	Tofacitinib	4	Case series	13	6	1	6	-	1 (7.7%)	8 (69.2%)	4 (30.8%)	NA	Headache, URI, transient elevation in hepatic transaminases
Liu^101^	2019	Ruxolitinib	4	Case series	1	-	-	1	-	1 (100%)	-	-	NA	URI, weight gain, acne, easy bruising, fatigue^ǁ^
Putterman^102^	2018	Topical tofacitinib	4	Case series	11	1	4	6	-	3 (27.3%)	5 (45.4%)	1 (9%)	NA	Irritation
Bayart^103^	2017	Topical tofacitinib or topical ruxolitinib	4	Case series	6	1	2	3		1 (16.7%)	3 (50%)	2 (66.7%)	NA	None
Craiglow^104^	2016	Topical ruxolitinib	5	Case report	1	-	-	1	-	-	1 (100%)	-	NA	Minor decrease in WBC
Laser and Light Therapy														
Fenniche^105^	2018	308 nm excimer lamp + topical khellin	5	Case report	1	-	-	-	1	1 (100%)	-	-	None	Mild transient erythema
Al-Mutairi^106^	2009	308 nm excimer laser	4	Case series	11	9	2	-	-	5 (45.4%)	3 (27.3%)	3 (27.3%)	4 (50%)	Mild erythema, peeling
Al-Mutairi^107^	2007	308 nm excimer laser	4	Case series	4	4	-	-	-	-	1 (25%)	3 (75%)	NA	Mild erythema, peeling
Zakaria^108^	2004	308 nm excimer laser	4	Case series	1	1	-	-	-	-	1 (100%)	-	NA	Mild erythema, hyperpigmentation
Phototherapy														
Jury^109^	2006	NBUVB	4	Case series	6	NA	NA	NA	NA	-	1 (16.7%)	5 (83.3%)	NA	Erythema, blistering, anxiety
Ersoy-Evans^110^	2008	PUVA	4	Case series	10	3	4	3	-	2 (20%)			NA	Erythema, pruritus, burning
Yoon^111^	2005	PUVA + TT	5	Case report	1	-	-	1	-	1 (100%)	-	-	NA	None
Mitchell^112^	1985	PUVA	4	Case series	5	3	2	-	-	-	5 (100%)	-	3 (75%)	None
Claudy^113^	1983	PUVA	4	Case series	7	2	2	3	-	3 (42.8%)	-	4 (57.1%)	NA	Pruritus
Amer^114^	1983	PUVA	4	Case series	2	1	1	-	-	-	-	2 (100%)	NA	None
Lux-Battistelli^115^	2015	PUVA + zinc	4	Case series	1	-	1	-	-	-	1 (100%)	-	1 (100%)	Seborrheic dermatitis, acne
Majumdar^116^	2018	Topical psoralen + natural sunlight	4	Case series	5	4	-	1	-	-	5 (100%)	-	NA	Erythema, irritation, hyperpigmentation, scaling
Belezos^117^	1965	UV irradiation + topical estrogen	4	Case series	1	1	-	-	-	1 (100%)	-	-	NA	None

*AA*, Alopecia areata; *AO*, alopecia ophiasis; *AT*, alopecia totalis; *AU*, alopecia universalis; *CR*, complete response; *GI*, gastrointestinal; *IMC*, intramuscular corticosteroids; *IV*, intravenous; *LoE*, level of evidence; *MTX*, methotrexate; *N*, number of patients; *NA*, not available; *NBUVB*, narrow-band ultraviolet B; *NR*, no response; *OC*, oral corticosteroids; *PDC*, pulse dose corticosteroids; *PR*, partial response; *PUVA*, psoralen ultraviolet A; *RR*, relapse rate; *SE*, side effects; *TC*, topical corticosteroids; *TT*, topical tacrolimus; *URI*, upper respiratory infection; *UV*, ultraviolet; *WBC*; white blood cells; *ZBC*, zinc biotin, and clobetasol.

* Complete response defined as ≥95% hair regrowth, (n %) = percent of total number of patients.

† Partial response defined as <95% and >0% hair regrowth, (n %) = percent of total number of patients.

‡ No response defined as 0% hair regrowth, (n %) = percent of total number of patients.

§ Relapse rate defined as number of patients who responded to treatment and experienced recurrence of hair loss, (n %) = percent of responsive patients.

|| Adverse events reported in both adult and pediatric patients.

¶ Patient(s) discontinued study due to adverse events.

# Study listed under multiple sections due to inclusion of multiple treatments.

**Table III.** Included studies evaluating miscellaneous treatment of alopecia areata in pediatric patients

First author	Year	Treatment	LoE	Study type	N	AA	AT	AU	AO	CR*	PR^†^	NR^‡^	RR^§^	SE
Liu^4^	2017	Apremilast	4	Case series	1	-	-	1	-	-	-	1 (100%)	NA	Diarrhea, nausea, headaches, lethargy
Cho^5^	2010	Botulinum Toxin A	4	Case series	3	-	1	2	-	-	-	3 (100%)	NA	None
Sarifakioglu^6^	2006	Topical sildenafil	4	Case series	8	-	-	-	-	-	3 (37.5%)	5 (62.5%)	NA	None
Fessatou^7^	2003	Gluten-free diet	4	Case series	2	-	-	-	-	1 (50%)	1 (50%)	-	NA	None
Boonyaleepun^8^	1999	IVIG	5	Case report	1	-	-	1	-	-	1 (100%)	-	NA	None
Shibuya^9^	1990	Bone marrow transplant^||^	5	Case report	1	-	1	-	-	1 (100%)	-	-	NA	Chronic GVHD skin eruption
Rozin^10^	2003	Cotrimoxazole	5	Case report	1	1	-	-	-	1 (100%)	-	-	1 (100%)	None
Zawahry^11^	1973	Aloe	4	Case series	1	1	-	-	-	-	1 (100%)	-	NA	None
Skurkovich^12^	2005	Anti-IFN gamma antibodies	4	Case series	16	11	5		-	-	12 (75%)	4 (25%)	1 (8.3%)	None
Willemsen^13^	2006	Hypnosis^¶^	4	Case series	2	-	-	2	-	1 (50%)	-	1 (50%)	1 (100%)	None
Letada^14^	2007	Topical imiquimod	5	Case report	1	-	-	1	-	-	1 (100%)	-	1 (100%)	None
Koblenzer^15^	1995	Psychotherapy^#^	5	Case report	1	1	-	-	-	-	1 (100%)	-	NA	None
Putt^16^	1994	Massage, relaxation, and reward	5	Case report	1	1	-	-	-	-	1 (100%)	-	NA	None
Teshima^17^	1991	Psychotherapy (PT) + OC and CYA vs OC and CYA	3	Case-control	5	-	-	5	-	PT + OC and CYA - 2/2 (100%); OC and CYA −1/3 (33.3%)		PT + OC and CYA - 0/2 (0%); OC and CYA - 2/3 (66.7%)	NA	None
Arrazola^18^	1985	Topical nitrogen mustard	4	Case series	4	2	2	-	-	-	4 (100%)	-	NA	Allergic contact dermatitis

*AA*, Alopecia areata; *AO*, alopecia ophiasis; *AT*, alopecia totalis; *AU*, alopecia universalis; *CR*, complete response; *CYA*, cyclosporin; *DPCP*, diphenylcyclopropenone; *GVHD*, graft-versus-host disease; *ILC*, intralesional corticosteroids; *IFN*, interferon; *IVIG*, intravenous immunoglobulin; *LoE*, level of evidence; *N*, number of pediatric patients; *NA*, not available; *NR*, no response; *OC*, oral corticosteroids; *PR*, partial response; *PT*, psychotherapy; *RR*, relapse rate; *SE*, side effects.

* Complete response defined as ≥95% hair regrowth, (n %) = percent of total number of patients.

† Partial response defined as <95% and >0% hair regrowth, (n %) = percent of total number of patients.

‡ No response defined as 0% hair regrowth, (n %) = percent of total number of patients.

§ Relapse rate defined as number of patients who responded to treatment and experienced recurrence of hair loss, (n %) = percent of responsive patients.

|| Postoperative cyclosporin and short-term methotrexate were also given for graft-versus-host disease prophylaxis.

¶ Both patients were simultaneously treated with selective serotonin reuptake inhibitors.

# Psychotherapy was supplemented by minoxidil and anthralin.

## CAPSULE SUMMARY

- Numerous therapies have been used to treat children and adolescents with alopecia areata (AA) with variable efficacy.
- Topical corticosteroids have the highest level of evidence for the treatment of pediatric AA, followed by contact immunotherapy. More clinical trials and comparative studies are needed to further guide management of pediatric AA.

## Abbreviations used:

AA: alopecia areata
AO: alopecia ophiasis
AT: alopecia totalis
AU: alopecia universalis
DPCP: diphenylcyclopropenone
LAD: lymphadenopathy
LoE: Levels of Evidence
PRISMA: Preferred Reporting Items for Systematic Review and Meta-Analyses
RCT: randomized controlled trial
SADBE: squaric acid dibutyl ester
